# Using gold nanoparticles to detect single-nucleotide polymorphisms: toward liquid biopsy

**DOI:** 10.3762/bjnano.11.20

**Published:** 2020-01-31

**Authors:** María Sanromán Iglesias, Marek Grzelczak

**Affiliations:** 1 Centro de Física de Materiales CSIC-UPV/EHU and Donostia International Physics Center (DIPC), Paseo Manuel de Lardizabal 5, 20018 Donostia-Sebastián, Spain; 2 Ikerbasque, Basque Foundation for Science, 48013 Bilbao, Spain

**Keywords:** amplification reactions, biomarkers, colorimetric biosensing, gold nanoparticles, plasmonics, single-point mutation

## Abstract

The possibility of detecting genetic mutations rapidly in physiological media through liquid biopsy has attracted the attention within the materials science community. The physical properties of nanoparticles combined with robust transduction methods ensure an improved sensitivity and specificity of a given assay and its implementation into point-of-care devices for common use. Covering the last twenty years, this review gives an overview of the state-of-the-art of the research on the use of gold nanoparticles in the development of colorimetric biosensors for the detection of single-nucleotide polymorphism as cancer biomarker. We discuss the main mechanisms of the assays that either are assisted by DNA-based molecular machines or by enzymatic reactions, summarize their performance and provide an outlook towards future developments.

## Introduction

Cancer is a leading cause of death accounting for about 8.8 million deaths in 2015 [1]. The list of tumor-linked substances, i.e., biomarkers for diagnosis and prognosis purposes is continuously increasing. Cancer biomarkers are present in tumor tissues or serum and encompass a wide variety of molecules, including DNA, mRNA, enzymes, metabolites, transcription factors, and cell surface receptors. The first report on cell-free DNA in body fluids by Mandel and Metais in 1948 [2], opened the possibility to screen the presence of a disease through a simple blood test, setting thus a milestone of “liquid biopsy”. Liquid biopsy has the potential to accelerate the early cancer diagnosis by the detection of biomolecules such as cell-fee DNA directly in blood samples.

Currently, the development of liquid biopsies is directly linked to the state-of-the-art of advanced techniques in the field of genomics such as digital PCR, next generation sequencing (NGS), fluorescence in situ hybridization (FISH) and BEAMing. These facilitate the fast design of mutational profiles of tumor DNA, helping the prioritization of anti-cancer therapy. Although these techniques are without parallel in the analysis of genetic material and the detection of mutations, they require an operation by specialized personnel in large infrastructures such as hospitals or research centers. The democratization of liquid biopsy and therefore the advancement of personal medicine needs efficient point-of-care devices that are simple to use (preferentially colorimetric), disposable and cost-efficient, making them available to a wide range of users. It has been shown that progress in the development of such devices requires improved strategies for signal transduction, which might rely on the use of emergent nanomaterials.

Over the last decade, a number of novel and optically active nanomaterials involving semiconductor or metal nanocrystals enabled the development of sensing devices with rather simple transduction mechanisms [3]. For example, the aggregation-induced color change of a solution containing plasmonic nanoparticles (from red to blue) in the presence of molecules offers an excellent tool for colorimetric sensing without the need of using advanced techniques. Similarly, selective fluorescence quenching of organic dyes or semiconducting nanoparticles by plasmonic nanoparticles offers an unprecedented sensitivity in native physiological media. Coupling these nanomaterial-based systems with enzymatic reactions can further increase the sensitivity and selectivity of a given sensor, leading to a scenario in which a tiny structural alteration of a biomolecule can be detected within seconds even at sub-picomolar concentration.

Here, we review recent advancements in the development of sensors based on metallic nanoparticles for the detection of mutations in circulating tumor DNA molecules. By introducing the importance of DNA molecules as biomarkers in the field of liquid biopsy and by discussing current technologies in clinics, we review the performance of recent sensors for single-point mutation in which gold nanoparticles act as signal transducers. We classify the discussed sensors according to whether the underlying mechanisms of detection involve enzymatic reactions or not.

## Review

### Liquid biopsy

Tissue biopsy is the state-of-the-art protocol in clinics for the evaluation of tumor progression. This procedure, however, constitutes a significant barrier for monitoring oncogenic mutations since it can introduce clinical risks for the patient [4], heterogeneity of tumor cells [5], and difficulties in the sampling of tumor cells that in turn can lead to inadequate amounts of tissue for genetic testing. Thus, the possibility of extracting valuable biochemical information on tumor progression directly from physiological media became a straightforward solution to the issues of conventional biopsy. Table 1 summarizes the main benefits of liquid biopsy concerning cancer diagnosis, prediction and prognosis emphasizing that it is a promising tool in monitoring tumor-specific changes during the entire course of the disease. It can be used for the early-stage detection of cancer, the identification of indicators for disease recurrence and progression and the evaluation of a given treatment in nearly real-time. In the context of the present review, the early-stage diagnosis of cancer by novel sensing devices is prioritized in the subsequent discussion.

**Table 1:** Benefits of liquid biopsy in diagnosis, prediction and prognosis of cancer.

stage	information/benefits	ref.

diagnosis	early detection	[6–11]
	monitoring of minimal residual disease	[12–15]

prediction	assessment of molecular heterogeneity of overall disease	[16–17]
	monitoring of tumor dynamics	[18–20]
	identification of genetic determinants for targeted therapy	[21–22]
	evaluation of early treatment response	[23–24]
	assessment of evolution of resistance in real time	[25–26]

prognosis	identification of high risk of recurrence	[27]
	correlation with changes in tumor burden	[28–29]

### Circulating tumor DNA and single-nucleotide polymorphism

**Figure 1:**
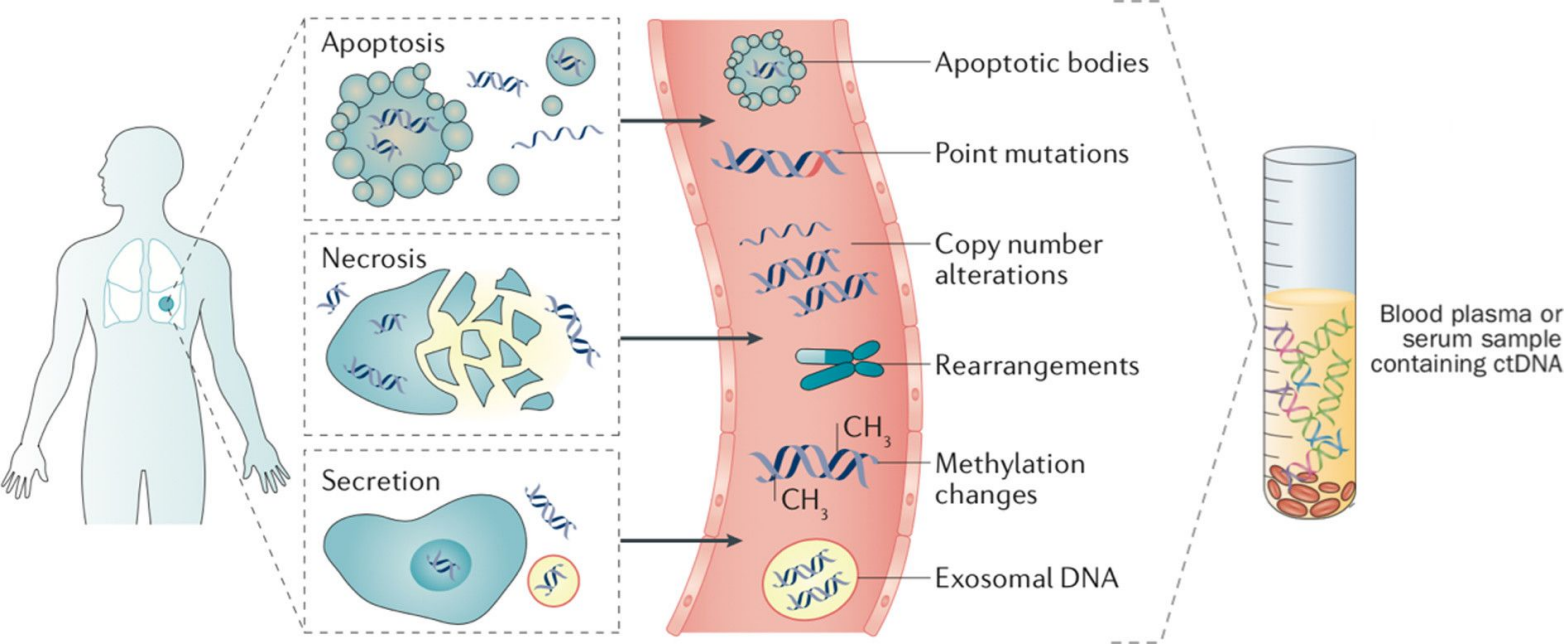
Alterations in cell-free DNA. Cell-free DNA can be released from both cancerous and normal cells located in the tumor environment through apoptosis, necrosis or secretion. Once in the bloodstream, cfDNA may exist either free or associated with extracellular entities such as exosomes. cfDNA can carry multiple classes of tumor-derived genetic alterations including point mutations, copy-number fluctuations and structural rearrangements. Reprinted with permission from [45], copyright 2017 Springer Nature.

The list of biomarkers that are present in blood and that exhibit potential for cancer diagnosis experiences continuously grows. These biomarkers include circulating tumor cells (CTCs) [30], circulating membranous structures [31], circulating cell-free nucleic acids (cfDNA) [4], microRNA, RNA [32] and proteins [33] (Figure 1). For the discussion here, the detection of circulating cell-free DNA is relevant. While all types of cells (tumor and nonmalignant) release cfDNA into the extracellular system [34], the circulating tumor DNA (ctDNA) is released uniquely by tumor cells. Several release mechanisms have been identified. 1) Secretion after cell death through apoptosis and necrosis, 2) secretion from tumor cells in the form of free or encapsulated DNA fragments, and 3) secretion from phagocytized tumor cells [35–38]. It has been observed that with the increase of tumor load, the local fraction of ctDNA increases compared to the overall amount of cfDNA in the sample [39]. However, this tendency is patient-dependent. The average length of ctDNA fragments generated from cell apoptosis ranges from 145 to 180 bp. Longer fragments of up to 10 kbp are secreted by cell necrosis [40–44].

**Figure 2:**
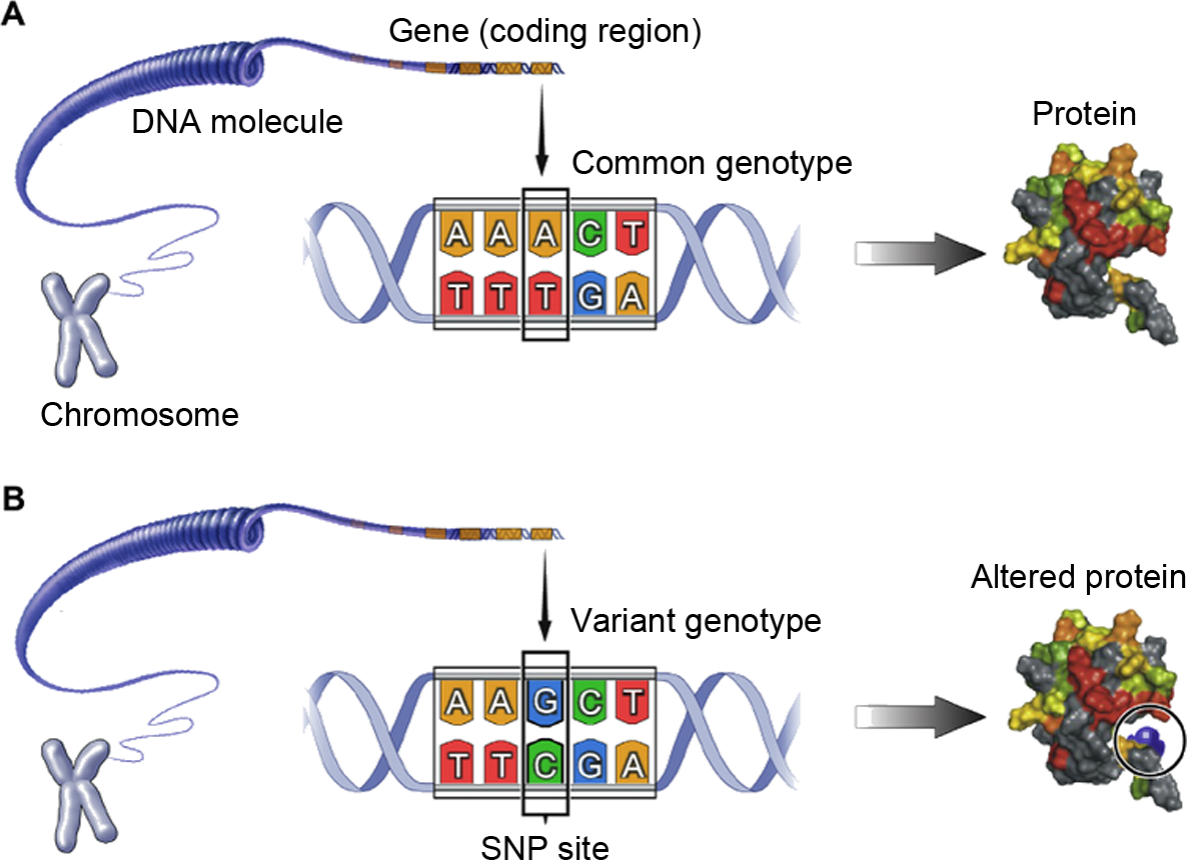
Single-nucleotide polymorphisms (SNPs) are genetic mutations that alter single base in DNA, causing sequence modification in amino acids and malfunction of a corresponding protein. Reprinted with permission from [51], copyright 2014 Elsevier.

Moreover, in solid malignancies, circulating tumor DNA differs from cell-free DNA by somatic mutations [18,46–47]. In leukemia, for example, the increased amount of cfDNA originates from cancer cells. Nonetheless, four main types of gene alterations occurring in cfDNA are classified:

1. **Single-point mutations or single-nucleotide polymorphism (SNP).** A base substitution at one nucleotide that may result in a change of the amino acid sequence of the encoded protein or premature truncation of the protein (Figure 2).
2. **Copy-number alteration.** Duplications, insertions or deletions of one or a few nucleotides leading to the addition or subtraction of amino acids in the protein.
3. **Exon or gene copy-number changes.** Large duplications or deletions encompassing entire exons (protein-encoding regions in a gene) and affecting the functional domains of the protein.
4. **Structural modifications.** Translocations or inversions within a gene that result in fusion genes and associated fusion proteins.

Among these four gene alterations, the SNPs in ctDNA are known as the major contributors to the genetic variations, representing more than 80% of all known polymorphisms at a frequency of around 1 every 1000 bases [48]. To date, around 1.42 million single-base variations have been identified. These small variations may occur in noncoding or coding regions of the genes. SNPs in coding regions can be either synonymous (without altering the encoded amino acid) or nonsynonymous (altering the encoded amino acid), hence, possibly altering the function of the corresponding protein (Figure 2) [49–50].

Approximately, 50% of all SNPs occur in noncoding regions, 25% are silent mutations with no effect on gene function and phenotype, and the remaining 25% lead to mutations of the gene function [52–53]. These SNPs can influence the promoter activity (gene expression), the activity of messenger RNA (mRNA), gene conformation (stability) and the translational efficiency. Keeping in mind the importance of these modifications, SNPs can be proposed as biomarkers in the clinical diagnosis of diseases, personalized medicine and drug treatment.

Genotyping is an essential process in determining which genetic variants alter the encoded amino acid sequence and thus the function of a given protein. Based on the molecular mechanism, the majority of SNP genotyping assays fall in one of four groups [54–55]. These include (1) allele-specific hybridization (mutation-dependent hybridization of short nucleotides with variable target DNA), (2) primer extension (DNA polymerase-based incorporation of specific desoxyribonucleotides complementary to the DNA template, (3) allele-specific ligation (ligase-based covalent linking of two oligonucleotides upon hybridization on a DNA template), and (4) invasive cleavage (nuclease-based cleavage of the 3D structure formed when two overlapping oligonucleotides hybridize perfectly to a target DNA). The genotyping method should meet several requirements, namely, facile development from sequence information, cost-efficiency, robustness of the reaction, scalability, high-throughput discrimination and the possibility for automatization with minimal hands-on operation. We will demonstrate in the following sections that the advancement of laboratory-based sensors for SNP discrimination benefits from the current state-of-the-art in genotyping techniques in SNP determination, especially in the context of conceptual novelty.

### Tumor-specific aberrations containing SNP

Because the fraction of circulating DNA that is derived from the tumor can range between 0.01% and 93% [41], analytical techniques of high sensitivity are currently implemented to obtain reliable information on tumor-associated genetic modifications and to follow tumor dynamics [4,16,46,56]. These techniques are mainly modifications of the well-known polymerase chain reaction (PCR), establishing thus the state-of-the-art in clinics in the discrimination of SNP. The most relevant types of cancer including colorectal, breast, ovarian, pancreatic and lung cancer comprise several common tumor-specific aberrations of single-point mutations, which are frequently selected as targets in the development of novel biosensors based on nanoparticles. Further below, we describe the working principles of biosensors that were designed for the detection of the primary tumor-specific aberrations listed in Table 2.

**Table 2:** Tumor-associated genetic modifications in circulating cell-free DNA.^a^

tumor type	tumor-specific aberration

colorectal cancer [18]	APC, KRAS, PIK3CA, TP53
breast cancer [19]	PIK3CA, TP53, BRCA1
ovarian cancer [56]	TP53, PTEN, EGFR, BRAF, KRAS
pancreatic cancer [57]	KRAS
non-small-cell lung cancer [58]	KRAS

^a^KRAS = Kirsten rat sarcoma; APC = adenomatosis polyposis soli; PIK3CA = phosphatidylnositol-4,5-bisphosphate 3-kinase catalytic subunit alpha; TP53 = tumor protein p53, BRCA1 = breast cancer gene 1, PTEN = phosphatase and tensin homolog, EGFR = epidermal growth factor receptor, BRAF = B-Raf proto-oncogene, serine/threonine kinase.

### Colloidal gold as a signal transducer in SNP detection

With the increased diversity of available optically active nanomaterials, optical assays have attracted wide interest. Particularly attractive is the colorimetric detection of analytes in a liquid phase, which represents a direct way to evaluate the presence of an analyte by the naked eye. This facilitates its implementation as a transduction system in point-of-care devices. Therefore, noble metal nanoparticles (metallic gold) are widely applied in the development of biological sensing devices. Gold is an inert metal that exhibits exceptional chemical stability in physiological media and the readiness for surface functionalization with desired biomolecules through stable Au–S bonds. The key properties of gold nanoparticles are their optical properties, which yield an exceptional light absorbance in the visible spectral range. This is explained by the fact that in the metallic core, the conductive electrons experience coherent oscillations in the presence of incoming electromagnetic radiation, thereby giving rise to the so-called localized surface plasmon resonance (LSPR). The position and the bandwidth of the LSPR can be modulated by the shape of the nanocrystals and can vary between 400 and 2000 nm. The high absorption cross section (plasmonic nanoparticles absorb photons over a region about ten times larger than their physical diameters) [59], and the lack of photobleaching (unlike organic fluorescent dyes and semiconductor nanocrystals) are additional parameters making plasmonic nanocrystals attractive materials for biosensing. Importantly, the position of the plasmon band and its bandwidth are also strictly related to the local environment. The collation of a nanoparticle at a nanometric distance from the surface of another nanoparticle induces a redshift of the maximum of the surface plasmon band because of plasmon coupling, causing a color change of the solution. Thus, the control over aggregation or redispersion processes is of paramount importance in the design of devices for the naked-eye detection of molecular events on the surface of nanoparticles.

**Figure 3:**
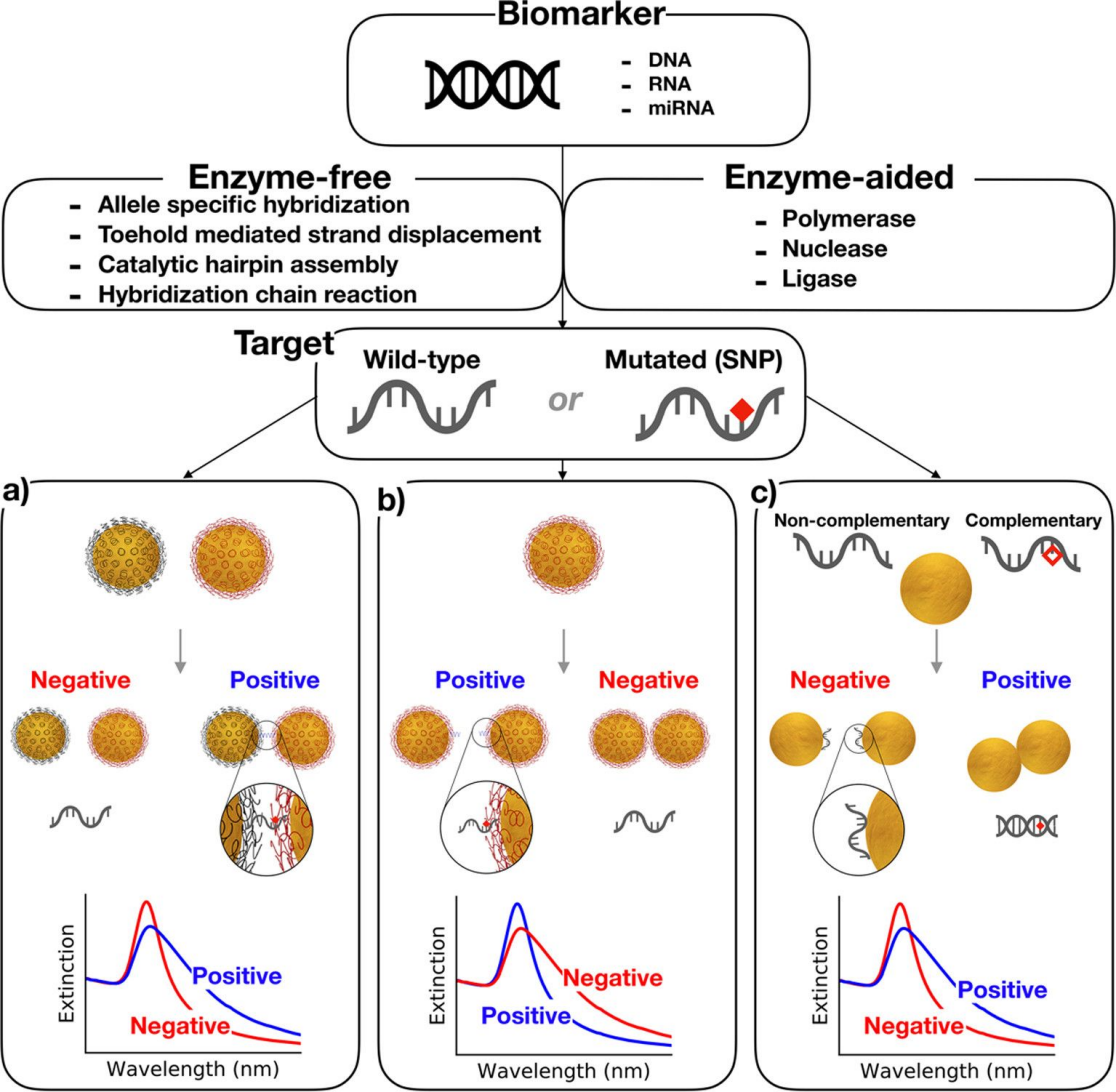
Gold nanoparticle-based colorimetric assays in the colloidal phase. a) Cross-linking hybridization assay: Through the specific hybridization of DNA, the distance between the particles decreases leading to a color change. b) Noncross-linking hybridization assay: An increase in the ionic strength causes an aggregation of nanoparticles (blue solution), which is prevented by the presence of the complementary target. c) Colorimetric assay based on unmodified nanoparticles: Single-stranded DNA (ssDNA) stabilizes gold nanoparticles against salt-induced aggregation, while in the presence of double-stranded DNA (dsDNA) particles undergo aggregation.

The early experiments by Mirkin and co-workers [60–61] on the aggregation of gold nanoparticles stabilized with radially distributed single-stranded DNA (Au@DNA) through selective hybridization of complementary DNA opened up new possibilities in the development of colorimetric sensors capable of discriminating single-point mutations. This methodology comprised the selective aggregation of two types of noncomplementary DNA-stabilized gold nanoparticles of 13 nm in diameter (Figure 3a). A target oligonucleotide (24–30 bases) that was complementary to the single-stranded DNA (ssDNA) of both types of nanoparticles induced an aggregation through hybridization. The main advantage of this method is the low risk of false positives.

Maeda et al. have proposed the so-called noncross-linking method for SNP detection, which is based on the nonselective aggregation of one single type of DNA-coated gold nanoparticles (Figure 3b) [62]. The target DNA (15 bases) containes a single-point mutation at the 5′ terminus, which provides steric stability to the nanoparticles, thus ensuring colloidal stability at a higher salt concentrations. On the contrary, the perfect match sequence (mutation-free 5′ terminus) formed a rigid double-stranded DNA (dsDNA) on the particle surface, decreasing steric and electrostatic repulsions, thereby causing gradual aggregation. This method allows for a sensitivity of 500 nM in the discrimination of single-base mutation.

Rothberg and Li proposed the use of citrate-stabilized gold nanoparticles instead of DNA-coated nanoparticles, which are prone to aggregate at a high salt concentration [63]. The working principle of this system rests on the fact that ssDNA exhibits a higher affinity to the surface of metallic gold as dsDNA. This is because ssDNA molecules contain a large number of functional groups facilitating electrostatic interactions with the gold surface (Figure 3c). Therefore, at a high salt concentration, adsorbed ssDNA stabilizes the nanoparticles against aggregation. In contrast, the preferential hybridization of target DNA with complementary strands left the nanoparticles uncovered, facilitating aggregation. The authors were able to detect single-base mismatches at the level of 43 nM using gold nanoparticles of 13 nm. This method, however, had a rather low selectivity and could only differentiate DNA strands containing three or more mismatched bases.

These examples are characterized by their simplicity requiring no expensive equipment or reagents. The detection of SNPs was achieved by mixing a DNA target with a probe followed by the direct naked-eye readout. The need for further improvements motivated the researchers to increases the complexity of the assays. Today, the methods for SNP detection can be classified as enzymatic and nonenzymatic. In enzyme-free strategies, the signal amplification is achieved by a cascade of hybridization reactions prior to the optical signal transduction. One of the mechanisms that are discussed in the following section benefits from free energy driving cascades of toehold-mediated strand-displacement reactions. Other heavily exploited mechanisms are based on the formation of DNA circuits and include hybridization chain reaction (HCR), catalytic hairpin assembly (CHA) and entropy-driven catalysis [64]. These mechanisms have shown a great potential for developing biosensors of high sensitivity and high selectivity since the target DNA itself is used as a catalyst to cyclically amplify the process of DNA self-assembly. The strategies based on enzymatic tools, on the other hand, are characterized by an extraordinary potential for signal amplification. The main advantages of using enzymes involve: a) the capability of modifying oligonucleotides (e.g., polymerases, nucleases, helicases, ligases), b) an extraordinary catalytic activity and c) biocompatibility. Table 3 summarizes the examples for the detection of DNA targets with single-point mutations using gold nanoparticles as signal transducers. We classified the assays by the used enzymatic reactions for signal amplification.

**Table 3:** Summary of particle-based optical assays for the detection of biomarkers.

nanoparticles (NPs)	target length/biomarker	procedure	limit of detection [Ref.]

**absorbance/enzyme-free**			

AuNPs (13 nm)	30 nt/—	hybridization assay	10 fmol [60]
AuNPs (13 nm)	24 nt/—	colloidal stability of NPS in the presence of ssDNA or dsDNA	100 fmol [63]
AuNPs (13 nm)	24 nt/—	hybridization assay	10 pmol [61]
AuNPs (13 nm)	14 nt/—	salt-induced aggregation of unmodified AuNPs	0.25 µM [65]
AuNPs (18 nm)	20 nt/EGFR	salt-induced aggregation of unmodified AuNPs	80 µM [66]
AuNPs (13 nm)	41 nt/JAK2	salt-induced aggregation of unmodified AuNPs	0.2 µM [67]
AuNPs (13 nm)	22 nt/—	a logic gate using two distinct target DNA molecules as input to discriminate SNPs using unmodified gold nanoparticles as indicators	100 pmol [68]
AuNPs (10 nm)	34 nt/—	the product of the catalytic strand displacement cascade disassembly AuNPs	0.1 µM [69]
AuNPs (13 nm)	14 nt/—	hybridization assay based on Au@LNA/DNA chimeras	0.1 µM [70]
AuNPs (15 nm)	15 nt/—	noncross-linking hybridization assay	0.5 µM [62]
AuNPs (14 nm)	395 nt/M. tuberculosis	noncross-linking aggregation of Au@DNA within rpoB locus	30 µg/mL [71]
AuNPs (14 nm)	16 nt/—	aggregation of unmodified AuNPs induced by CHA	0.1 pM [72]
AuNPs (40 nm), microbeads (MBs, 2.8 μm)	101 nt/KRAS	MBs@streptavidine hybridizes with the biotinylated target that is complementary to Au@DNA	20 pM [73]
AuNPs (13 nm)	22 nt/—	hybridization of peptide nucleic acid (PNA) and DNA prevents aggregation of nanoparticles	1 µM [74]
AuNPs (13 nm)	24 nt/CFTR	hybridization assay using a miniaturized optical monitoring system	10 nM [75]
AuNPs (15 nm)	22 nt/—	sequential hybridization to the target by allele-specific surface-immobilized capture probes and gene-specific Au@DNA	500 ng genomic DNA [76]
AuNPs (15 nm)	24 nt/—	hairpin-based amplification assay combined with lateral flow test	10 pM [77]
AuNPs (13, 20, 40 nm)	24 nt/—	aggregation of AuNPs by target-induced DNA circuits	200 pM (HCR), 14 pM (CHA) [78]
AuNPs (30 nm), MBs (2–3 μm)	27 nt/—	MBs@DNA, Au@DNA and target hybridization followed by magnetic separation and scanometric detection based on silver reduction for signal amplification	500 zM (10 copies) [79]
AuNPs (13 nm)	27 nt/—	sandwich assay between target, Au@DNA and a flatbed scanner; signal amplification by Ag reduction	50 fM [80]
AuNPs (20 nm)	84 nt/EGFR	hairpin assembly produces short DNA catalyst, which induces aggregation of unmodified AuNPs	7.7 fM [81]
AuNRs	24 nt/—	combination of HCR and unmodified gold nanorods for signal transduction	1.47 nM [82]
AuNPs (13 nm)	19 nt/BRCA1	DNA-fueled molecular machine modulates the kinetics of Au@DNA aggregation	0.26 nM [83]
AuNPs (15 nm)	38 nt/—	target DNA hybridizes with Au@DNA, triggering a HCR that inhibits aggregation of AuNPs	0.5 nM [84]
AuNPs (43 nm)	22 nt/—	oriented aggregation of nanoparticles on Y-shaped DNA duplex	10 pM [85]
AuNPs (40 nm), MBs (1.5 μm)	30 nt/—	MBs@DNA, Au@DNA, target hybridization followed by magnetic separation and scanometric detection based on silver reduction for signal amplification	100 amol [86]
AuNPs (15 nm)	265 nt/hepatitis C	release and adsorption of free primers on the nanoparticle ensuring stability	50 copies [87]
AuNPs (75 nm)	22 nt/—	hybridization assay	3 nM [88]
AuNPs (5, 10, 12, 20 nm)	60 nt/KRAS	target-stabilized nanoparticles interacting with matching or mismatching probe lines in a microfluidic channel	5 fmol [89]
AuNPs (13 nm), growth (40 nm)	20 nt/—	DNA hybridization-mediated autocatalytic overgrowth of gold nanoparticles	60 nM [90]
AgNPs (13 nm)	22 nt/—	PNAs induce aggregation of citrate-stabilized AuNPs, which is prevented by DNA targets that complex selectively to PNA	1 µM [91]
AuNPs (18 nm)	12, 21, 42 nt/c-KIT	selective aggregation of PNA-stabilized AuNPs by target DNA and positively charged AuNPs through electrostatic interactions	0.1 µM [92]
AuNPs (15 nm)	30 nt/hepatitis A	combination of “click chemical” ligation chain reactions on gold nanoparticles and a magnetic separation to detect DNA and RNA	50 zM [93]
AuNPs (13, 46, 63 nm)	19 nt/BRCA1	colorimetric detection based on sandwich assay	10.85 fmol [94]
AuNPs (63 nm)	70, 140 nt/EGFR	colorimetric detection based on sandwich assay combined with a preincubation step	100 pM [95]
AuNPs (25, 53 nm)	70, 140 nt/EGFR	chemical modifications of capture probes for a selective aggregation of nanoparticles	5 nM [96]
SiO_2_ microparticles (MPs) and AuNPs	22 nt/miR-21	DNA I located on SiO_2_MPs captures miRNA, DNA II labeled with EDTA·2Na chelates Au^3+^ ions and regulates the growth of AuNPs	8.9 fM [97]

**absorbance/enzyme-aided**			

AuNPs (56, 13 nm)	30 nt/—	modulation of the enzyme activity of thrombin on the surface of AuNPs relative to fibrinogen	12 pM [98]
AuNPs (13 nm)	112, 230, 316 nt/BRCA1	allele‐specific PCR with thiol-labeled primers for the specific stabilization of unmodified AuNPs	20 ng genomic DNA [99]
AuNPs (13 nm)	40 nt/KRAS	selective ligation of two adjacent Au@DNA probes in the presence of a mutation	74 pM [100]
AuNPs (14 nm)	36, 48, 80 nt/—	nanoparticle amplification assisted by nicking endonuclease (NEase) for the detection of target DNA	10 pM [101]
AuNPs (15 nm)	24 nt/keratin 10	isothermal strand displacement polymerase reaction to produce biotin–digoxin-labeled dsDNA in combination with a lateral flow strip	0.08 pM [102]
AuNPs (20 nm)	40 nt/—	aggregation of Au@DNA in the presence of single-strand-specific nuclease	100 nM [103]
AuNPs (13 nm)	22 nt/miR-122	miRNA–probe heteroduplex as substrate of double strand nuclease, releasing a probe to aggregate the nanoparticles	16 pM [104]
AuNPs (13 nm)	43 nt/EGFR	coupling of invasive reactions with NEase-assisted nanoparticle amplification to produce linkers that prevent aggregation	1 pM [105]
AuNPs (12 nm)	33 nt/—	exonuclease III (Exo III)-induced cleavage of dangling ends on Au@dsDNA causing specific aggregation	2 nM [106]
AuNPs (13 nm)	23 nt/—	coupling of polymerase and NEase through an isothermal exponential amplification reaction to selectively detach DNA from Au@DNA	46 fM [107]
AuNPs (13 nm)	30 nt/KRAS	cyclic enzymatic cleavage based on Exo III in the presence of the target and a linker to induce aggregation of Au@DNA	15 pM [108]
AuNPs (15 nm), MBs (1 μm)	46 nt/BRCA1	Au@DNA complexed with magnetic beads using horseradish peroxidase (HRP, enzymatic catalysis) and bovine serum albumin (BSA, nonspecific blocker)	25 pM [109]
AuNPs (13 nm)	34 nt/—	combination of padlock probe for rolling-circle amplification and NEase-assisted nanoparticle amplification	1 pM [110]
AuNPs (42 nm)	1130 nt/ chlamydia trachomatis	isothermal target and probe amplification for the hybridization of target amplicons and Au@DNA followed by RNA cleavage	10^2^ copies [111]
AuNPs (15 nm)	16, 32 nt/ cytochromes P450	single‐base primer extension in combination with noncrosslinking assay	1 μM [112]
AuNPs (13 nm)	16–80 nt/rtM204V	selective stabilization of unmodified AuNPs with nucleoside monophosphates after nuclease cleavage	5 nM [113]
AuNPs (20 nm)	—/hepatitis B	ligation chain reaction to induce aggregation of Au@DNA	20 aM [114]

**fluorescence/enzyme-free**			

AuNPs (13 nm)	26 nt/—	combination of AuNP fluorescence anisotropy and toehold-mediated strand-displacement reaction	0.95 nM [115]
PS NPs (40 nm)	57 nt/PKD1	fluorescence-enhancement from nanoparticle-hybridized DNA complexes by nanostructured photonic crystals due to phase matching of excitation and emission	50 aM [116]
Ag nanoclusters	22 nt/miR-141	target-triggered CHA and fluorescence enhancement of DNA–silver nanoclusters to detect miRNA	0.3 nM [117]
AuNPs (5 nm)	24 nt/—	distance-dependent modulation of electrochemiluminescence from CdS:Mn nanocrystals by gold nanoparticles.	2.9 fM [118]
quantum dots (QDs, 10 nm)	19 nt/miR-21	p19-QDs with multiplex antenna capture miRNA-21/antimiRNA-21-Cy3 duplex to form a Förster resonance energy transfer (FRET) detection system between QDs and Cy3	0.6 fM [119]
MBs (1 μm)	21 nt/miR-27a	dual toehold-mediated circular strand displacement amplification-based DNA circuit as fluorescent strategy for the detection of miRNA-27a	0.8 pM [120]

**fluorescence/enzyme-aided**			

AuNPs (5 nm)	25 nt/—	enhancement of the electrochemiluminescence of a CdS film by a combination of an isothermal circular amplification reaction of polymerase, NEase and Au@DNA	5 aM [121]
QDs (15 nm)	21 nt/miR-196a2T	miRNA detection by coupling rolling circle amplification and NEase with streptavidin-coated QDs	51 aM [122]
carbon dots (CDs, 8 nm)	52 nt/H7N9 virus	carbon nanodot‐based fluorescent sensing strategy for H7N9 utilizing isothermal strand displacement amplification	3.4 fM [123]
graphene QDs (5 nm)	22 nt/miR-141	pentaethylenehexamine- and histidine-functionalized graphene QD for a microRNA fluorescence biosensing nanoplatform coupled with molecular beacon double-cycle amplification	0.43 aM [124]
QDs (5 nm)	—/miR-148, miR-21	QD-assisted FRET signal generation	42 fM [125]

**optical microscopy**			

AuNPs (5 nm)	22 nt/LET7	differential interference contrast microscopy with a microarray platform comprising hairpins as capture probes and Au@DNA as signaling probe	10 fM [126]
AuNPs (40 nm)	45 nt/p53	aggregation of oligonucleotide-modified organic nanospheres coded with fluorescent dyes (red/green/blue)	200 fmol [127]
AuNPs (15 nm)	15 nt/—	surface plasmon resonance imaging of Au@DNA in a PDMS–gold–glass hybrid microchip	32 nM [128]
AuNPs (50 nm)	28, 60, 90 nt/—	dark-field microscopy for the detection of head-to-tail Au@DNA hybridization	4 pM (28 nt)/40 pM (60 nt) [129]
AuNPs (50 nm)	46 nt/HeLa cells	dark-field microscopy combined with Rayleigh scattering spectrophotometry for single-particle detection	10 HeLa cells [130]

### Enzyme-free SNP discrimination using gold nanoparticles

Although the first works on the selective aggregation of Au@DNA by complementary ssDNA offered a conceptual novelty in the field of biosensing, the simple aggregation of nanoparticles via complementary target DNA suffered from low detection sensitivity and selectivity. To overcome these limitations, new amplification methods were proposed using diverse molecular mechanisms. Mirkin and co-workers have implemented a bio-bar-code method to detect single-point mutations in target DNA associated with the anthrax factor [79]. The assay comprised magnetic beads coated with a sequence of oligonucleotides and Au@DNA modified with two types of oligonucleotides (1:100 ratio), one that was complementary to the target sequence and the other complementary to a bar-code sequence. The magnetic beads and the Au@DNA formed a sandwich structure linked by the target sequence, which was magnetically separated from the wild-type DNA. This process was followed by the release of bar-code sequences, which were then captured on a chip-based scanometric system and amplified via the autocatalytic reduction of silver(I) by hydroquinone. The authors demonstrated a sensitivity of 500 zM, which translates to about ten copies in 30 μL of the sample.

To improve the sensitivity of the colorimetric sensors, several groups have proposed to use modified nucleic acids. The most common modified nucleic acids are peptide nucleic acids (PNAs) and locked nucleic acids (LNAs), which are obtained by intercalating natural and artificial nucleobases or by modifying internucleoside linkages. Lee and co-workers have proposed the colorimetric detection of a point mutation in codon 559 of c-Kit using targets of different lengths (12-, 21- or 42-mer) [92]. PNA-coated AuNPs (18 nm) were complementary to the wild-type target sequence that hybridized with the PNA probes on the AuNPs. This hybridization made the particles negatively charged because of the phosphate backbone in the target DNA sequences preventing their aggregation. Uncharged particles underwent aggregation. The detection limit was 100 fmol/μL for a target with 21 bases. Chakrabarti and Klibanov built an assay based on the difference in the thermal stability of PNA–DNA and DNA–DNA duplexes [131]. The authors observed that the PNA-modified AuNPs aggregated due to the neutralization of the charges of the PNAs. Upon the addition of complementary DNA, the colloidal stability was recovered because of the hybridization of the negatively-charged DNA with the neutral PNA-modified AuNPs. The group of Graham has reported the functionalization of AuNPs (13 nm) with LNAs, revealing a remarkable binding affinity and selectivity towards DNA targets with 22 bases [70]. The use of LNA/DNA chimeras enhanced the stability of duplexes formed with AuNP conjugates, which could discriminate between mismatching DNA and complementary target DNA at a detection limit of 100 nM.

The use of unmodified gold nanoparticles as signal transductors is another strategy for the selective detection of SNPs. Zhou and co-workers have proposed the use of binary DNA probes that were split in the middle and complementary to the target DNA [67]. Upon the addition of target DNA to the solution of binary DNA probes and citrate-protected AuNPs of 13 nm in diameter, the hybridization between the target DNA and the binary DNA probes enhanced the salt-induced aggregation. However, the presence of DNA molecules with single-base mismatches prevented the aggregation of AuNPs and the solution remained stable. This approach yielded a detection limit of 5 nM. Lee and co-workers have reported the detection of mutations in exon 19 and exon 21 of the epidermal growth factor receptor (EGFR) isolated from both the lung cancer cell lines and the cancer tissues of patients with non-small-cell lung cancer [66]. The citrate-stabilized gold nanoparticles underwent selective aggregation upon the addition of mutated DNA that hybridized with the complementary probe of 20 bases. Yet, the gold nanoparticles remained stable in the presence of wild-type DNA complementary to the probe sequence. In the eight specimens of non-small-cell lung cancer patients, the deletion of the mutant form of exon 19 and the L858R point mutation in exon 21 were detected at a concentration of 10 ng/μL.

Another strategy to improve the sensitivity of colorimetric biosensors based on plasmonic nanoparticles is the use of a molecular tool known as toehold-mediated strand displacement [132], in which one strand of DNA (output) is exchanged spontaneously with another strand (input). In such a process, an original ssDNA strand, which is complexed with a protector ssDNA, has a region – the toehold – that is complementary to the third strand of an ssDNA – the invading strand. The displacement starts with the selective hybridization of the invading strand with original strands, followed by a progressive branch migration of the invading domain to finally displace the protector ssDNA. The process is energetically favored since the reverse reaction is slower by several orders of magnitude. When the protector strand possesses a toehold region, it can turn into an invading strand itself, giving rise to a strand-displacement cascade. Duan et al. have proposed the use of a toehold-mediated strand-displacement cascade, in which the product of the strand displacement (length of 34 bases) was consumed by the disassembly of AuNPs [69]. The assay allowed for an SNP discrimination at a detection limit of 1 nM in a complex physiological medium such as fetal bovine serum.

**Figure 4:**
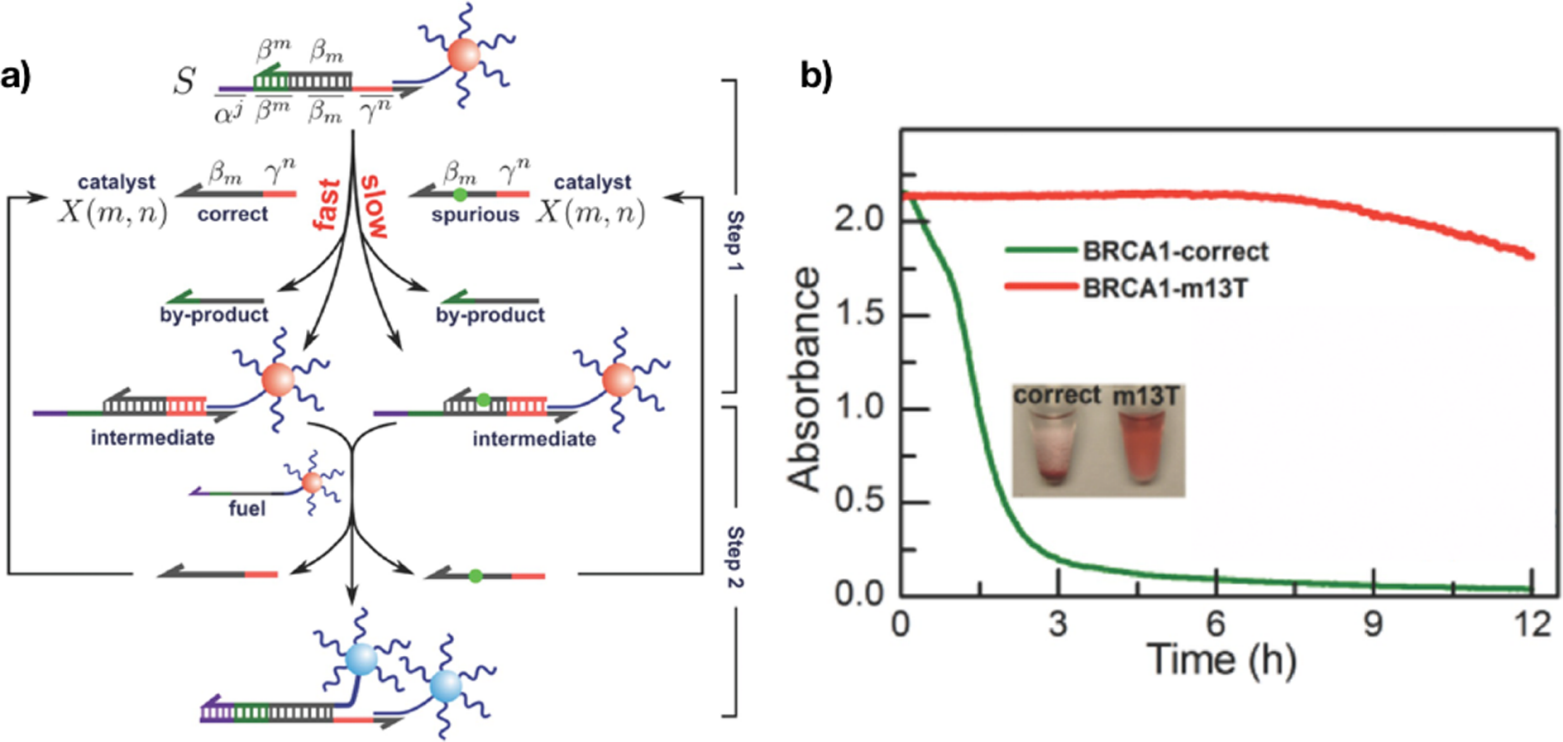
Discrimination of SNPs by means of the kinetics of particle aggregation. a) The spurious catalyst decelerates the toehold-exchange reaction and thus the formation of aggregates. (b) Time-dependent change of the color of the assay using spurious and correct targets. Reprinted with permission from [83], copyright 2014 John Wiley and Sons.

Liang and co-workers [83] built on the previous results and developed a strategy to discriminate single-base mutations through the assembly of AuNPs driven by a DNA-fueled molecular machine (Figure 4). In their design, the initial mixture contained two different types of nanoparticles: first, AuNPs functionalized with multi-stranded DNA molecules (S), and second, Au@DNA acting as the fuel. The key in the design of S were two open-terminal domains, named α and γ. A reaction was initialized by a catalyst (X) binding to the γ domain of S. Thus, releasing a by-product, an intermediate species was produced, which had a single-stranded region complementary to the DNA sequence of the fuel. In the next step, the fuel displaced the catalyst X, resulting in the cross-linking aggregation of the two DNA–AuNP complexes. The catalyst was released back into the solution. The authors observed that the aggregation of nanoparticles was slower by a factor of 10 when the sequence of the catalyst contained single-base mismatches (spurious catalyst). This colorimetric bioassay could detect single-base changes associated with the breast cancer gene BRCA1. The detection limit was 0.26 nM, corresponding to 31.2 fmol of a target.

Recently, advances in the development of DNA circuits based on hybridization and strand-exchange reactions have attracted much attention. Two examples of DNA circuits are commonly exploited, HCR [133] and CHA [134], both introduced by the Pierce group. These are based on the storage of potential energy in two hairpin species. In HCR, a single-stranded DNA initiator interacts with the first hairpin, exposing a new single-stranded region, which in turn opens the second hairpin species. Then, the chain reaction proceeds. Namely, single-stranded regions that are identical to the original single-stranded DNA initiator get exposed and finally open other hairpins. As a result, double helices are formed until all hairpins are consumed. On the other hand, CHA relies on the exponential amplification of a target sequence. In the presence of a target, the so-called hairpin detection probe (HDP) is unfolded to form a duplex and expose its concealed domain. The corresponding hairpin assistant probe (HAP) replaces the target to form specific HDP/HAP complexes. The target is released based on a thermodynamically driven entropy gain process. The released target then triggers the next cycle to produce numerous HDP/HAP complexes [135].

In their work, Sang and co-workers [72] have proposed a method called target-catalyzed hairpin assembly amplification. Aggregation of nanoparticles at an elevated salt concentration was prevented by three kinetically frozen hairpin structures, which exhibited an affinity toward metallic gold. The addition of a DNA target activated a cascade of assembly steps to form stiff branched junctions, thus consuming all three hairpin structures. The electrostatic repulsion between the junctions and the negatively charged AuNPs made negligible their binding to the gold surface, leading to the aggregation at high salt concentrations. By this method, a detection limit of 0.1 pM for a single-point mutation in sequences of 16 nucleotides was achieved.

**Figure 5:**
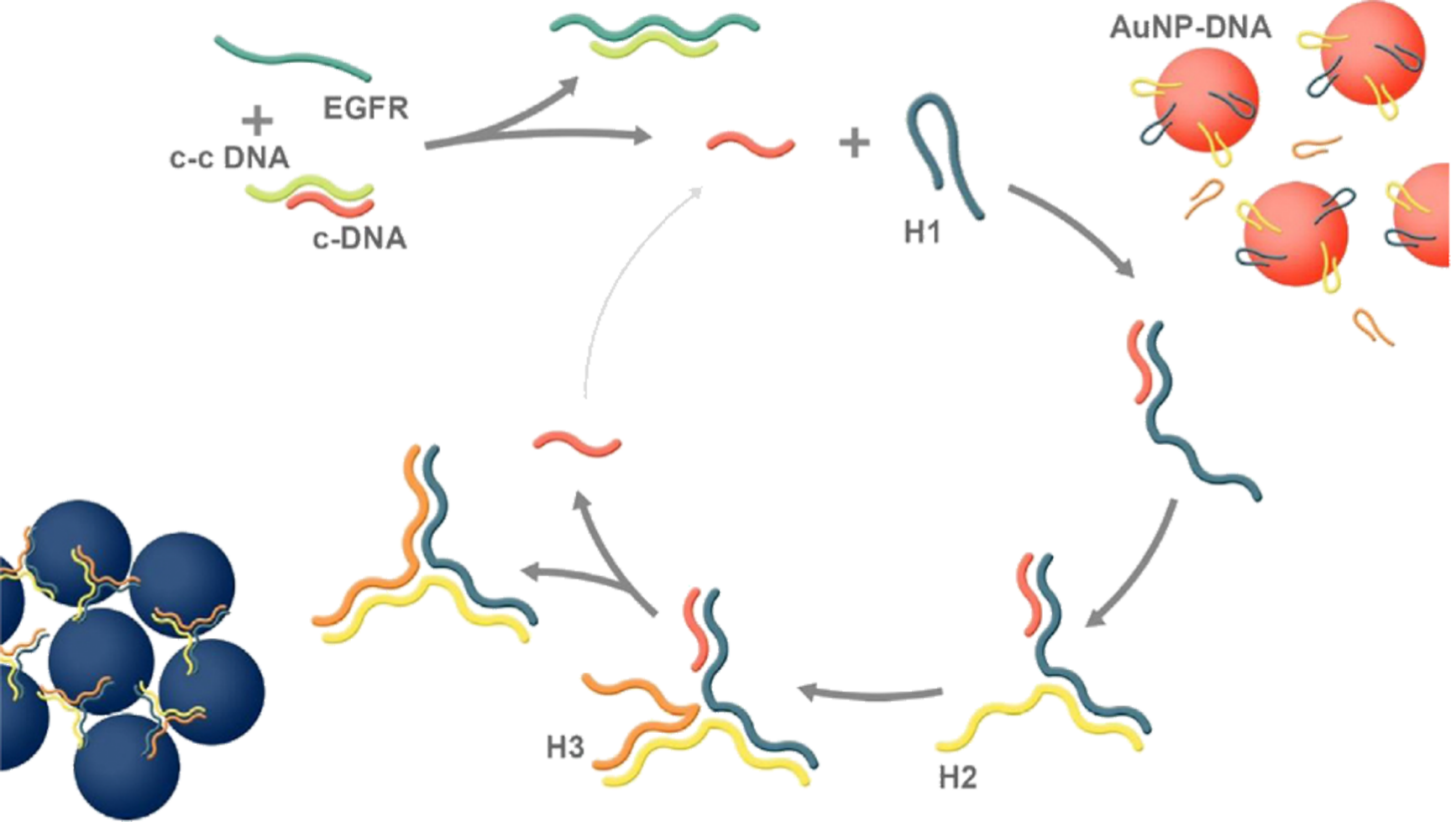
Working principle of the colorimetric assay for the detection of EGFR mutants in long DNA sequences. The presence of a target releases the catalyst oligonucleotide initiating CHA, which in turn progressively aggregate gold nanoparticles. Reprinted with permission from [81], copyright 2018 John Wiley and Sons.

Chanho Park et al. [81] have extended this strategy by using catalyst DNA (c-DNA) to discriminate single-base mutations in long (84 molecules) EGFR mutated DNA. The catalyst c-DNA was complementary to the so-called c-c DNA, a longer DNA sequence (Figure 5). The introduction of target DNA to a solution containing c-DNA and c-c DNA led to the formation of duplexes between c-c DNA and target DNA molecules. As a result, c-DNA was released again and initiated the assembly of branched junctions with three metastable hairpin DNA molecules. Two of these hairpins were attached to the surface of AuNPs, such that the formation of the branched junctions altered the colloidal stability of the nanoparticles, leading to a gradual aggregation. The detection limit of this method was 7.7 fM.

**Figure 6:**
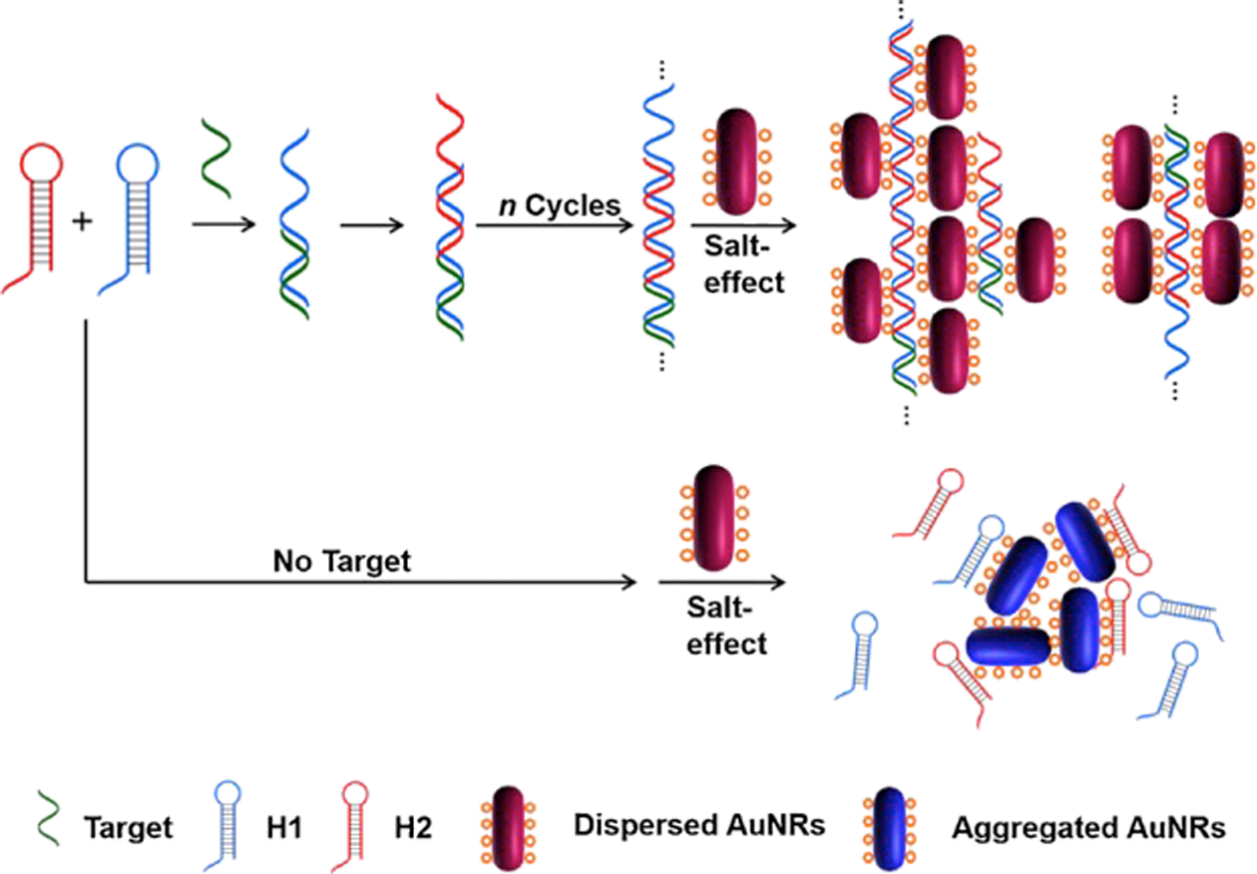
The combination of unmodified gold nanorods as signal transducers in an HCR amplification process for the colorimetric detection of single-base mutations. Reprinted with permission from [82], copyright 2018 Elsevier.

With the aim of improving the sensitivity of the plasmon-based colorimetric sensor, Ying and co-workers [82] have used gold nanorods as a signal transducers. Due to their intense longitudinal surface plasmon band, gold nanorods exhibit a higher sensitivity to changes of the colloidal stability as compared to spherical nanoparticles. Thus, by using gold nanorods one can lower the detection limit. The authors used unmodified gold nanorods for an HCR process as illustrated in Figure 6. The presence of a target DNA induced the hybridization of a hairpin DNA, producing a nicked double helix. Through electrostatic repulsion, this double helix prevented the aggregation of the gold nanorods at high salt concentrations. However, in the absence of a target DNA, the gold nanorods easily aggregated because of weak protection by hairpin DNA. Following this approach, the authors were able to detect target DNA in a range of 0–60 nM at a detection limit of 1.47 nM. The method is highly selective in distinguishing matching and single-base mismatching DNA.

### Enzyme-aided SNP discrimination

Owing to their high selectivity and strong catalytic properties, enzymes are molecular tools capable of improving sensitivity and selectivity of colorimetric sensors by several orders of magnitude. The most commonly exploited enzymes are nucleases, polymerases and ligases. Another advantage of using enzymes is their capability to sustain catalytic cycles, thus amplifying the number of available oligonucleotide sequences, which after reaching a concentration threshold induce the aggregation of nanoparticles. Therefore, a relatively low concentration of enzymes is required (1 U/µL) to maintain cyclic reactions and amplify the concentration of the DNA strands. However, the time required to reach the concentration threshold of a given strand is relatively long (hours), slowing down the operation of an assay. An additional drawback is that enzymatic reactions require a precisely controlled temperature range to ensure proper functioning, affecting the robustness of an assay under real-world conditions. Nonetheless, as shown over the last decade, the integration of enzymes into nanoparticle-based colorimetric assays is a conceptual innovation in the discrimination of DNA mutations in complex physiological media.

**Figure 7:**
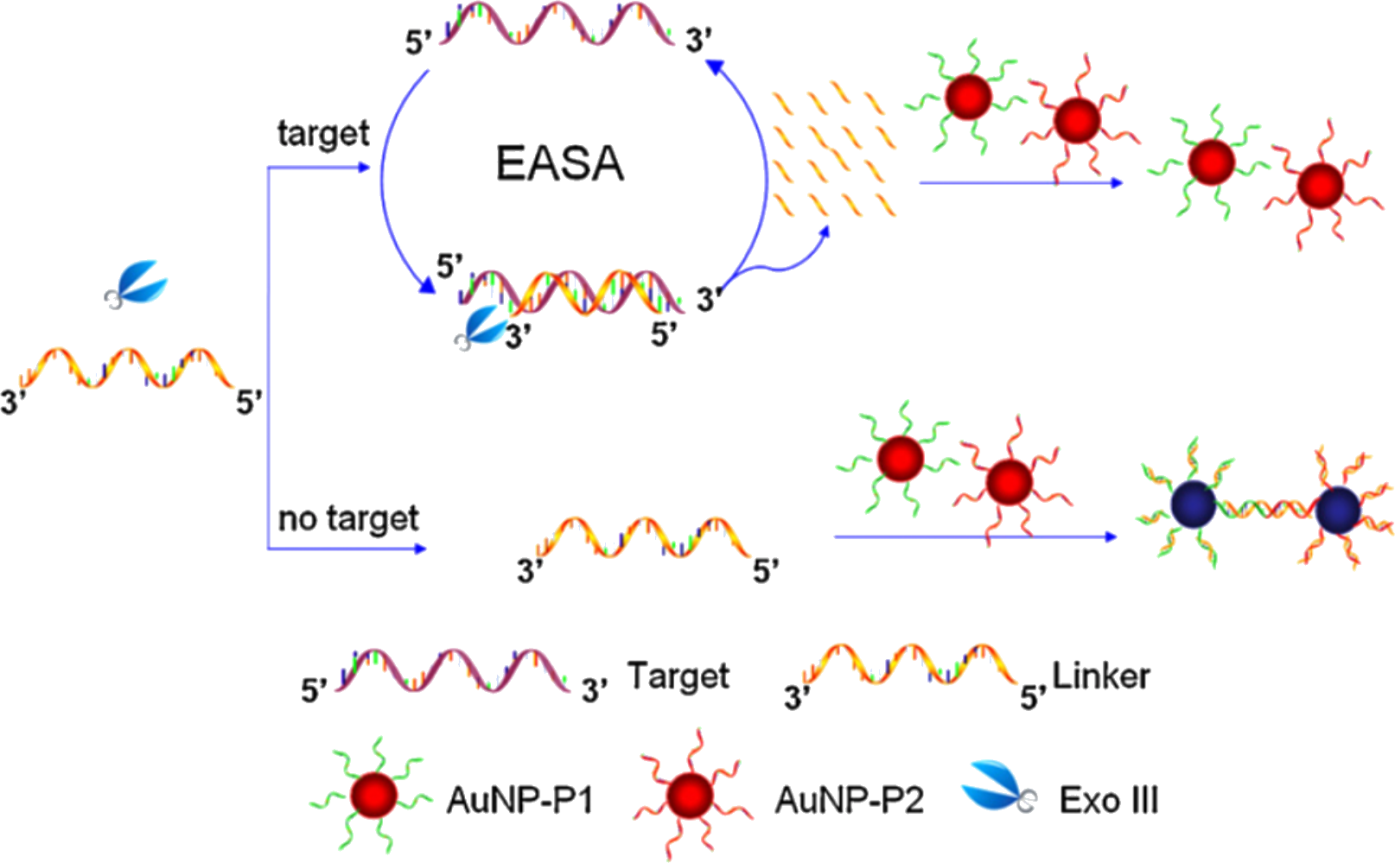
Working principle of EASA for the colorimetric detection of DNA mismatches. The consumption of a large amount of linker DNA strands by a few initial target molecules leads to the preservation of the colloidal stability of gold nanoparticles. In the absence of a target, the intact linker DNA hybridizes with Au@DNA leading to a gradual color change. Reprinted with permission from [108], copyright 2011 Elsevier.

To implement enzymes into assays for single-nucleotide discrimination using gold nanoparticles as signal transducers, exonuclease-assisted signal amplification (EASA) is commonly used. By coupling cyclic enzymatic cleavage and signal amplification by gold nanoparticles, Yang and co-workers were able to reach a detection limit of 15 pM [108]. Their system consisted of exonuclease III (Exo III), a linker strand and two batches of spherical Au@DNA (Figure 7). The linker was hybridized with a target DNA forming a duplex that could be digested by Exo III, which in turn led to the release of the target for other binding/cleavage events. The linker sequence was complementary to Au@DNA. When digested by Exo III, the linker could not couple with the Au@DNA particles, thus preventing particle aggregation. However, in the absence of a target, no linker DNA was digested, allowing for the progressive hybridization to Au@DNA and therefore aggregation. The activity of Exo III strictly depends on the type of 3′ terminus affecting the selectivity of the assay. More precisely, a fully complementary target led to recessed 3′ termini, while a mismatch yielded protruding 3′ termini. Hence, the method allowed for the discrimination of single and triple-base mismatches.

**Figure 8:**
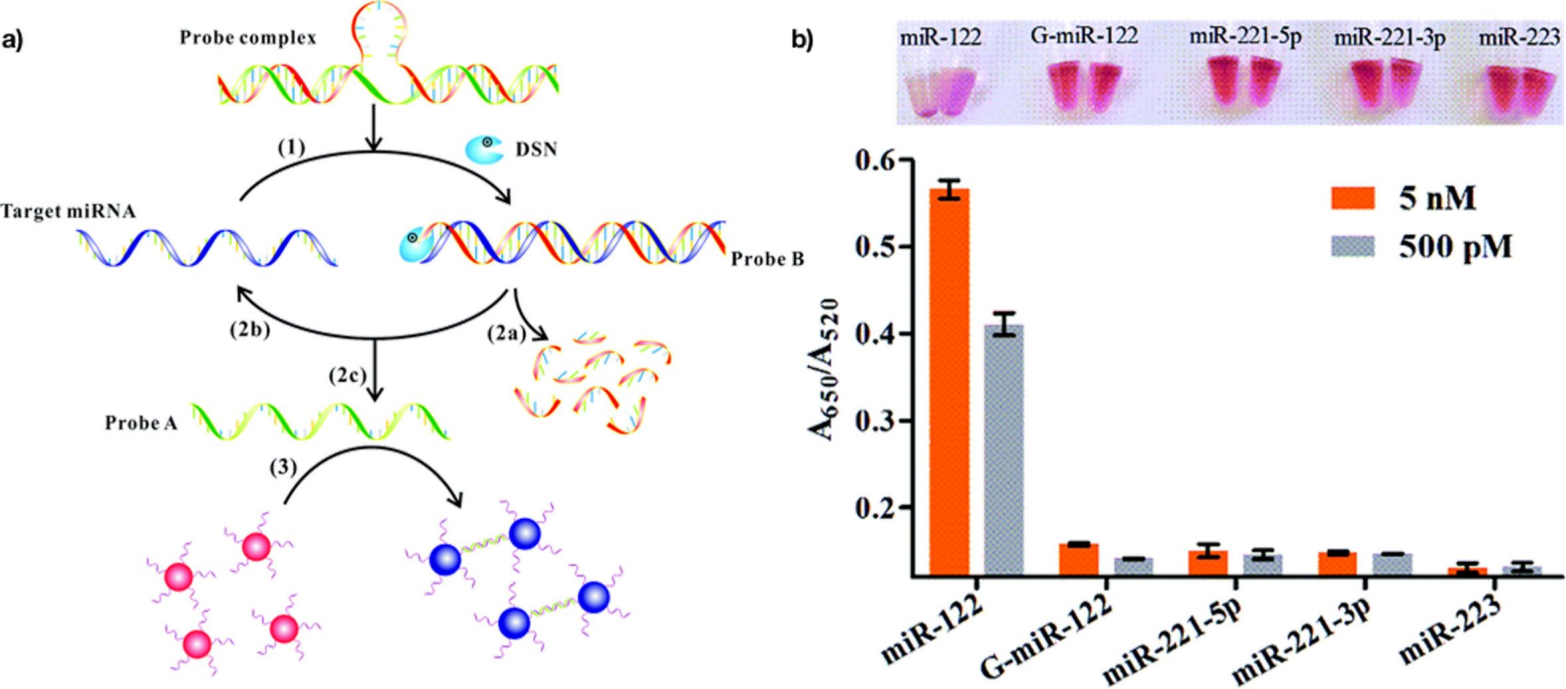
Schematic illustration of the colorimetric method for the detection of specific miRNA based on the amplification of DSN-assisted nanoparticles using AuNPs as the signal output. Reprinted with permission from [104], copyright 2015 Royal Society of Chemistry.

Ye and co-workers explored the duplex-specific nuclease (DSN) [104] for the detection of microRNA (Figure 8). The system consisted of a probe complex, DSN and two sets of different ssDNA-modified gold nanoparticles. The probe complex is formed by two strands with a loop in the middle and hybridization regions at the termini. Target miRNA invaded the probe complex forming a substrate (probe B) that was hydrolyzed by the enzyme, leading to the release of probe A and target miRNA. This cyclic invasion/digestion process caused a gradual increase of the concentration of probe A, triggering the aggregation of nanoparticles. Without a miRNA target, the probe complex remained undigested and the colloidal stability of the nanoparticles was conserved. DSN is capable of discriminating single-nucleotide mismatches in short DNA–RNA duplexes. The method reached a detection limit of 5 nM for mismatched miRNA (G-miR-122).

Another enzyme-assisted approach used in the discrimination of single-base mutations involves the combination of nicking endonuclease (NEase) and DNA polymerase giving rise to the so-called isothermal exponential amplification reaction (EXPAR). The EXPAR technique uses a DNA template, deoxynucleotide triphosphates (dNTPs) and two enzymes to achieve the exponential amplification of a target sequence. DNA polymerase and NEase are used in the same reaction mixture. Once connected to the template, the trigger DNA is extended along the template by the DNA polymerase. After the extension, the dsDNA contains the NEase recognition sequence, and the NEase cleaves the extended trigger DNA strand. The displaced strand and another template molecule then hybridize, initiating a new amplification cycle [136–137].

**Figure 9:**
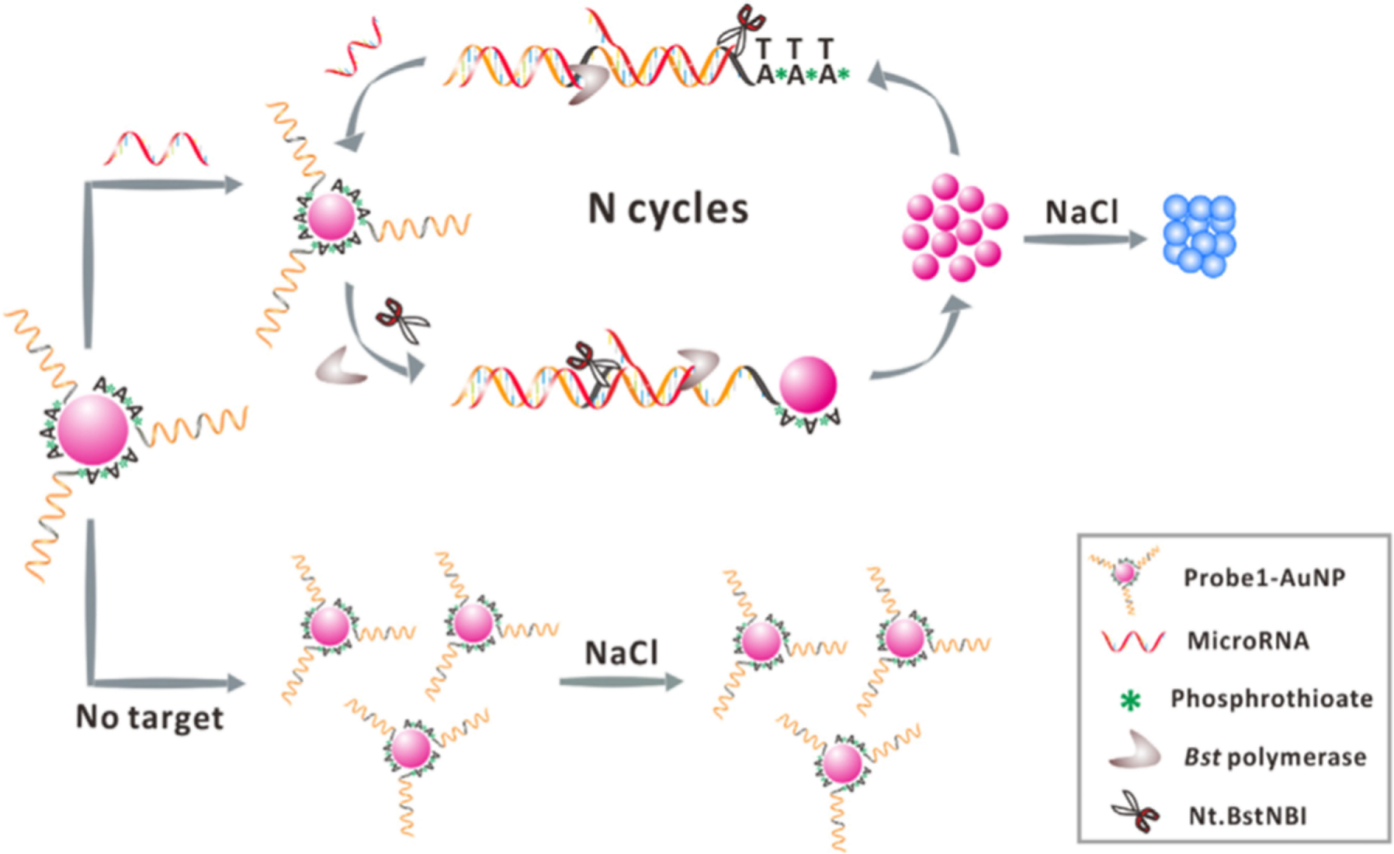
Colorimetric method for the detection of specific miRNA based on the combination of enzyme-assisted exponential amplification and AuNP-based colorimetric detection. The sensing probe attached to the gold nanoparticles can be easily disrupted by the enzymes, ensuring thus an improved performance. Reprinted with permission from [107], copyright 2016 Elsevier.

The group of Ye has used EXPAR with a sensing probe immobilized on the surface of gold nanoparticles (Figure 9) [107]. The key point here was the design of the sensing probe, which had three domains: a polyadenine block with a phosphorothioate modification in the backbone, two sequences complementary to the target miRNA and a recognition site of the NEase. When the target miRNA connected to the sensing probe, the EXPAR process commenced. The DNA polymerase extended the double-stranded fragment with a recognition site for an endonuclease that nicked the fragment enabling its release. This new fragment by being equal to the target miRNA, initiated a new EXPAR cycle, leading to the release of the sensing probe from the nanoparticles and as a consequence, their aggregation. This method yielded a detection limit of the specific miRNA of roughly 46 fM. In addition, the authors tested the selectivity of this assay using G-miR-221-3p. This test revealed a relatively good specificity, demonstrating that single-nucleotide differences between similar miRNAs members could be identified.

**Figure 10:**
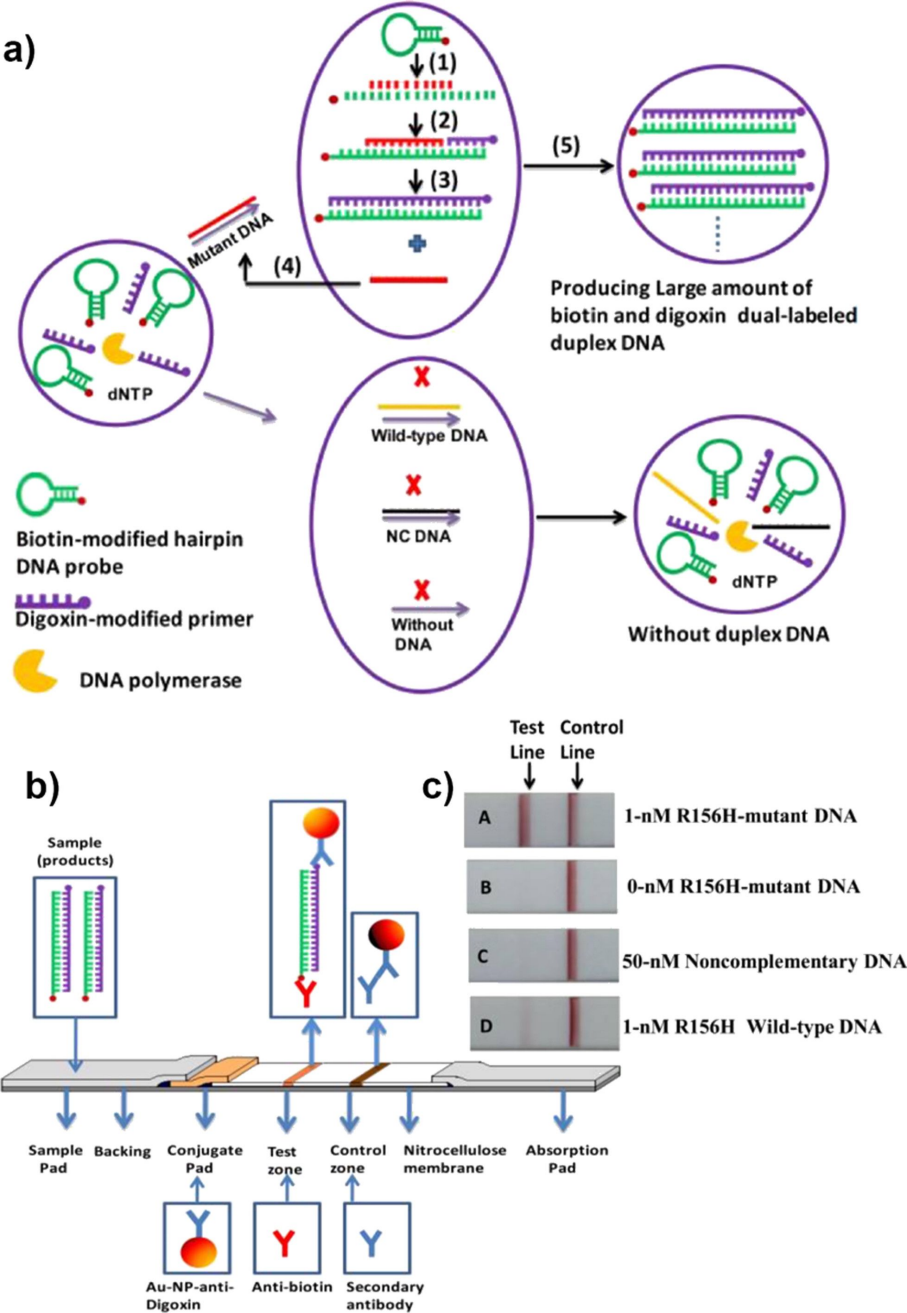
The combination of isothermal strand-displacement polymerase reactions and lateral flow strip for visual detection of gene mutations. a) Schematic illustration of isothermal strand-displacement polymerase reactions and the formation of digoxin- and biotin- attached duplex DNA complexes b) The mechanism of detection on lateral flow strip and c) visualization of the presence of mutation. Reprinted with permission from [102], copyright 2012 Elsevier.

To improve visual detection of single-point mutation on device conditions, Liu and co-workers developed an approach that combined isothermal strand-displacement polymerase reactions (ISDPR) and lateral flow strip (LFS) [102]. The mixture for ISDPR comprised biotin-modified hairpin, digoxin-modified primer, polymerase, and deoxynucleotide mixture (Figure 10a). A mutant DNA hybridized with the hairpin probe, leading to a conformational change, and stem separation (step 1), followed by polymerization reaction by the polymerase (step 2), forming biotin- and digoxin-attached duplex DNA (step 3). The process was further amplified through the next cycle (step 4). Overall, by the cyclic process, a large number of biotin- and digoxin-attached duplex DNA were produced using a little amount of the initial mutant DNA. The duplex DNA was later detected visually on the LFS through dual immunoreactions, using two batches of gold nanoparticles functionalized either with anti-digoxin and anti-biotin antibodies to obtain color signal at test and control zones, respectively. With this test, authors were able to detect visually the presence of R156H-mutant gene at the concentration down to 1 fM.

**Figure 11:**
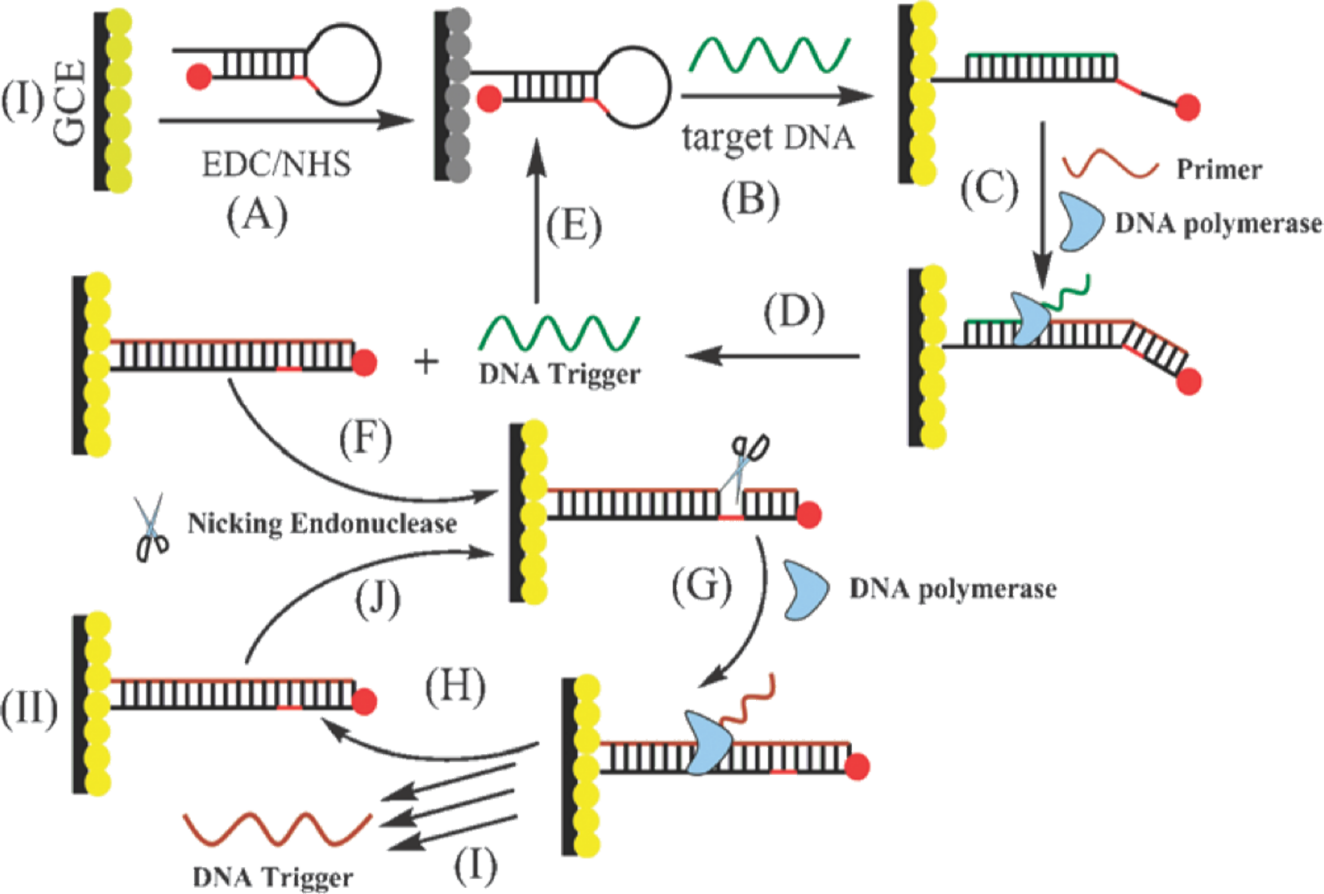
The use of gold nanoparticles as fluorescence quencher in the discrimination of SNP through cyclic enzyme-aided polymerization and nicking of oligonucleotides. The presence of a mutation decreases the fluorescence of the CdS films by nearly 90%. Reprinted with permission from [121], copyright 2011 Royal Society of Chemistry.

Another interesting approach of using gold nanoparticles for SNP detection is based on the fact that gold nanoparticles are capable of quenching fluorescence through Förster resonance energy transfer. By involving an isothermal circular amplification reaction of polymerase and NEase, the group of Chen [121] used gold nanoparticles to either quench or enhance the electrochemiluminescence of CdS films through the modulation of the distance between metallic and semiconductor components by a DNA machine (Figure 11). Their system consisted of a CdS film, a composite of AuNPs (5 nm) and hairpin DNA, a primer, and polymerase and NEase. In the presence of AuNPs–hairpin DNA, the luminescence of CdS was quenched (Figure 11A). The luminescence was recovered after adding target DNA, causing a conformational transition from hairpin to linear DNA. This opening initiated polymerization of DNA (Figure 11C), which in turn displaced previously hybridized target DNA (Figure 11D). In such a design, one target DNA strand could open many hairpin DNA strands, increasing the fluoresce signal. An additional increase of the fluorescence signal was achieved by the action of NEase that nicked an extended double helix strand, allowing for a subsequent polymerization process and displacement of the DNA trigger. The authors estimated that the presence of one polymerase molecule and one NEase molecule was enough to complete 22 cycles over a period of 40 min. The use of a target DNA with single-point mutation led to a decrease of the relative fluorescence by 87%.

**Figure 12:**
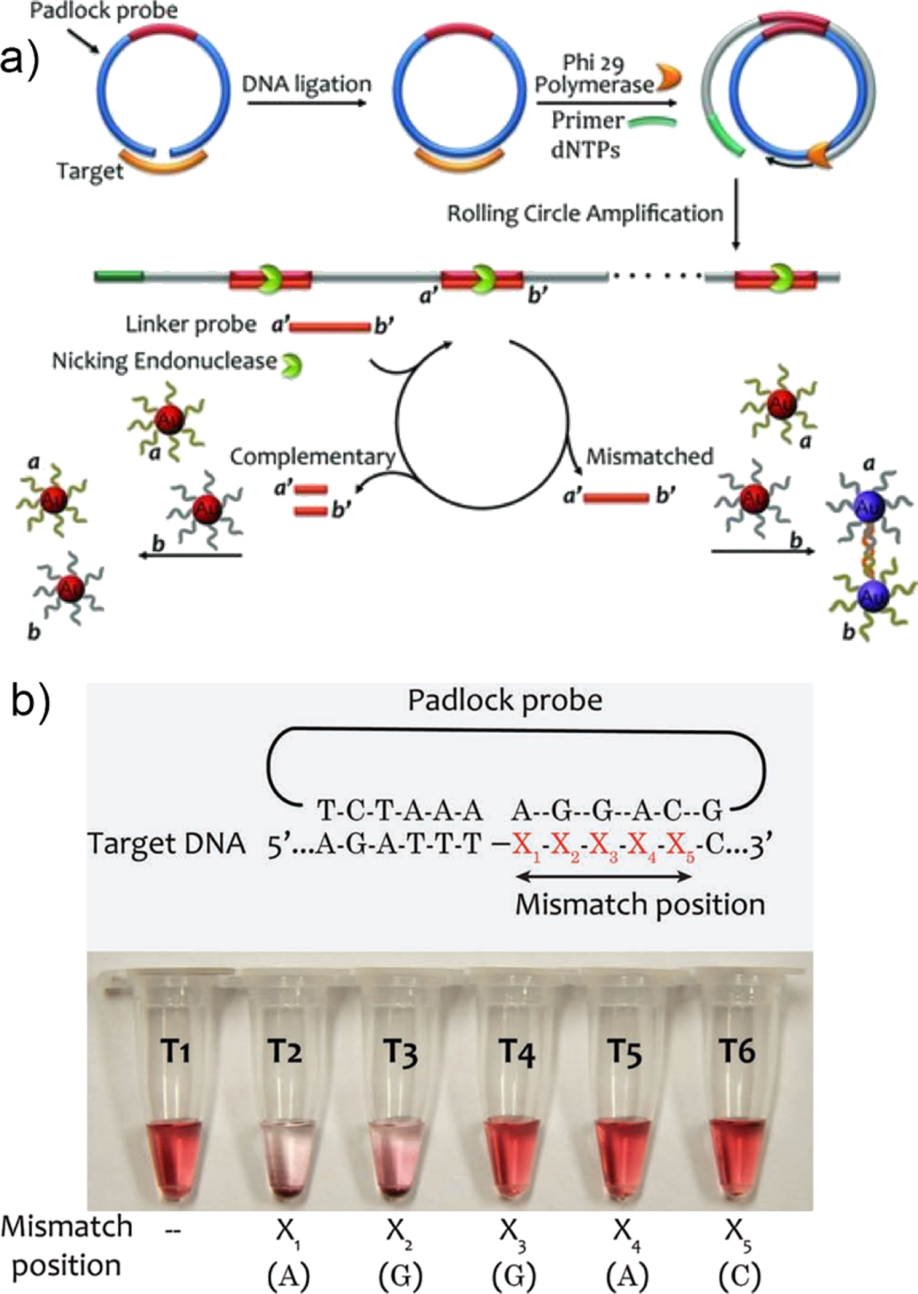
Colorimetric DNA detection through rolling circle amplification (RCA) and NEase-assisted nanoparticle amplification (NEANA). a) Working principle of the assay. b) Colorimetric detection of single-point mutation located in the proximity of ligation point. Reprinted with permission from [110], copyright 2012 John Wiley and Sons.

DNA ligase is an enzyme capable of repairing nicks, i.e., missing phosphodiester bonds, in a DNA sequence. Assays based on such enzyme rely on the ability to covalently join two oligonucleotides when they hybridize next to each other on a given template. In an inverted scenario, one can use the so-called padlock probe, a linear oligonucleotide with two terminal segments complementary to a short target sequence. Upon hybridization to a target DNA, the two ends become juxtaposed and can be joined by a DNA ligase if there is a perfect match. Liu and co-workers [110] have proposed the use of a padlock probe that contained a segment with the sequence identical to a linker probe *a′–b′* and target-complementary segments at both termini of the molecule (Figure 12a). The ends were brought in contact to form a circular oligonucleotide upon hybridization to the target DNA in the presence of a DNA ligase, serving as a template for a rolling circle amplification reaction. The produced prolonged DNA containing copies of the complementary sequence of the padlock template underwent selective scission in the presence of endonuclease. As a result, if the target DNA was complementary to the padlock probe, the nicked linker block would bind to the complementary DNA attached to gold nanoparticles without aggregation. If there was a mismatch, however, the linker probe would remain intact, facilitating the gradual aggregation of nanoparticles through hybridization. The authors demonstrated a colorimetric discrimination of single-point mutation, which decreases when the mismatch position is shifted away from the ligation site (Figure 12b).

**Figure 13:**
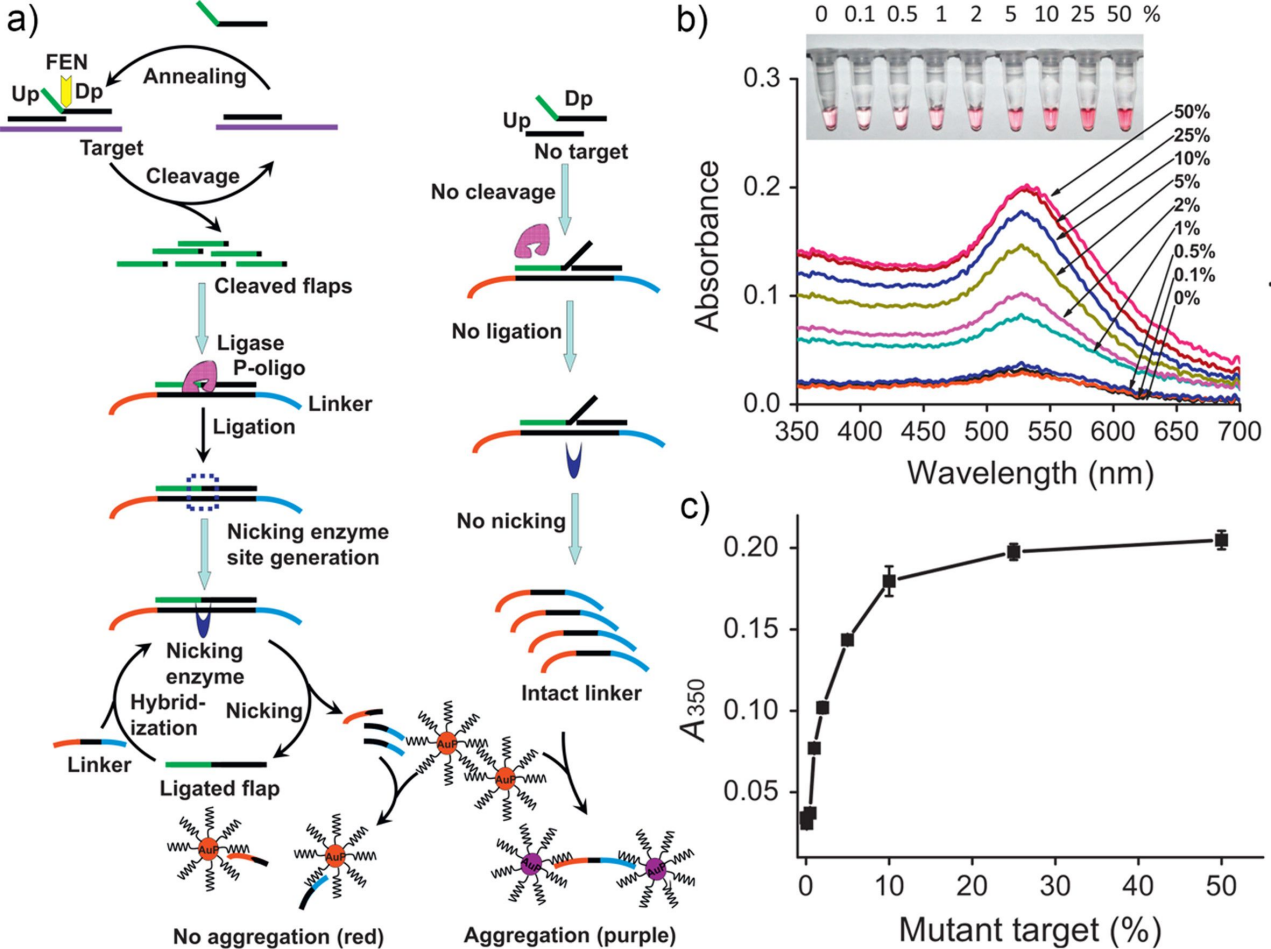
a) The working principle of DNA target detection through an invasive reaction coupled with NEase-assisted nanoparticle amplification. b) Optical characterization of the assay solution spiked with different amounts of c.2573 T>G mutant EGFR gene in the presence of a background wild-type EGFR sequence, showing a specificity down to 1%. c) The relation of increased absorbance with increasing the relative concentration of mutated sequence in assay mixture. Reprinted with permission from [105], copyright 2015 Elsevier.

In another study, Zhou and co-workers have proposed a colorimetric detection of DNA by coupling an invasive reaction (strand displacement) with NEase-assisted nanoparticle amplification [105]. In the proposed method, the target sequence was first hybridized to two probes (up- and down-stream) followed by enzymatic cleavage using flap endonuclease, producing many flaps from a target DNA (Figure 13a). Then, in another enzymatic reaction, the flaps were ligated with a P-oligo sequence, allowing for a nicking of the complementary strand (Linker) by NEase. The amplified linker strands bound to Au@DNA gold nanoparticles preventing their aggregation. In contrast, in the absence of the target, the consecutive enzymatic reactions were inhibited, leading to the preservation of the linker strands, and its subsequent hybridization with gold nanoparticles, causing aggregation. The specificity of the method was demonstrated by the discrimination of mutated strands (1%) in the presence of a large amount of wild-type DNA backgrounds (Figure 13b,c).

## Conclusion

The last two decades of extensive studies have proven that plasmonic nanoparticles (especially gold) exhibit properties that facilitate their implementation in molecular assays the for detection of genetic mutations in biological samples. Recent results also show that further complexification and coupling of the nanoparticles with DNA-based molecular amplification tools is a way to provide tests of binary readout and of rational sensitivity, with limits of detection reaching real-world requirements. It is, however, noteworthy that recent advancements, as discussed above, have been made mostly in the context of the (bio)molecular components of given assays. That is, while the amplification methods, based on DNA molecular machines or enzyme-assisted processes, were the subject of constant improvements, the plasmonic component remains barely explored. However, a vast number of plasmonic nanomaterials with different shapes, sizes and compositions is commonly available, offering a broad range of optical properties not only in the visible but also in the near-infrared spectral range. Shape anisotropy (rods) and regiospecific surface functionalization (tip versus lateral parts) enable the fabrication of colloidal systems with limited degrees of freedom. In such systems, the possible orientations of particles relative to each other are restricted, which imposes a colorimetric transition, i.e., a blueshift or redshift of the localized surface plasmon resonance [138]. It is reasonable to assume that development of biosensors for liquid biopsy will benefit from growing research on dynamic self-assembly of nanoparticles, in which interparticle forces [139], mutual orientation and interparticle distances are well controlled by chemical stimuli. Finally, we foresee that the detection of genetic mutations by plasmonic nanoparticles will be strongly enhanced by a complementary detection of disease-related proteins. Specifically, the recently proposed protein corona sensor arrays in which the composition of protein corona reflects the presence of a given cancer enabled new venues in detecting diseases directly from a blood sample [140]. Moreover, the simultaneous detection of genetic mutations and disease-specific proteins, as shown recently [141], brings great promises to liquid biopsy.

With the aim of reaching real-world applications, the current assays, which have demonstrated functionality under laboratory conditions, require optimization to be incorporated into point-of-care diagnostic tools. Lateral flow tests are especially attractive since they exhibit numerous advantages including minimal operator-dependent interpretation of the test (binary output), small value, low cost, relatively short detection time (minutes), and the possibility for integration with personalized electronic devices [142–145]. Still, the integration of different stages such as sample preparation, molecular amplification, and transduction zones requires new design of lateral flow devices to become fully operative. Recent works, as the one by Liu et al. discussed above, suggests the feasibility of such an approach [102].

Several limitations need to be addressed as well. One of the known issues in colloidal biosensing is the spontaneous formation of a protein corona on the surface of the particles in physiological media [146], inhibiting the interaction of the biomarkers with the colloid, thereby altering the sensitivity and selectivity of an assay. The inhibition of binding events by the corona layer may lead to false negatives, while corona-mediated unspecific binding leads to false positives. To minimize the protein adsorption, one can tailor the chemical composition of ligands by the use of zwitterionic compounds or the use of antifouling polymers such as polyethylene glycol.

Finally, the availability of whole-genome sequencing as offered by recently established private companies (e.g., Veritas Genetics and Novogene) allows for the determination of hundreds of mutations from a single sample at relatively low cost (200 US dollars). However, the time required to complete such an analysis exceeds several weeks, making it unfeasible for the fast monitoring of tumor dynamics. Therefore, point-of-care diagnostic tools that offer rapid (hours) discrimination of an individual mutation remain an aim to be pursued in the further development of personalized medicine.
